# Effects of dual-task training on attentional function among community-dwelling older adults: study protocol for a randomized controlled trial

**DOI:** 10.1186/s13063-026-09510-z

**Published:** 2026-02-06

**Authors:** Maki Ogasawara, Hiroshi Hayashi, Kazuaki Iokawa, Takaaki Fujita, Koshi Sumigawa, Hironori Kawamata, Iori Kawasaki, Daisuke Matsumoto, Toshimasa Sone

**Affiliations:** Department of Occupational Therapy, School of Health Sciences, Fukushima Medical University, 10-6 Sakae-Machi, Fukushima City, Fukushima, 960-8516 Japan

**Keywords:** Attentional function, Healthy older adults, Dual-task training, Randomized controlled trial, Protocol

## Abstract

**Background:**

Attentional function is the basis of cognitive function, and its decline affects the daily lives of older adults. Previous studies have not consistently reported the effects of dual-task training (DTT) on attentional function in community-dwelling older adults. This study aims to verify the effectiveness of DTT by combining “motor tasks” and “cognitive tasks involving motor activity” with a focus on inducing dual-task interference (DTI).

**Methods:**

The study design is a randomized controlled trial. The intervention consists of DTT that combines “motor tasks” involving lower limb movements with “cognitive tasks involving motor activity” incorporating complex finger movements. The program will be implemented in a DTI setting, and tasks will be adjusted individually for each participant. The intervention group will be conducted twice per week for four weeks, with each session lasting one hour. The control group will continue with the participants’ usual daily activities for four weeks. Attentional function will be assessed as the primary outcome using the Trail Making Test-Japanese and as secondary outcomes using the digit span test and the Stroop and reverse-Stroop test. Balance function will be measured using the single-leg stance test. All evaluations will be conducted at baseline and post-intervention. In the statistical analysis, paired *t*-tests will be used to compare pre-intervention and post-intervention changes within each group, and analysis of covariance will be used to compare intervention effects between groups.

**Discussion:**

Based on the study objectives, the maintenance and improvement of attentional function should be promoted to help community-dwelling older adults maintain healthy lives in familiar environments.

**Trial registration:**

UMIN, UMIN000057681. Registered on 30 June 2025. UMIN website https://center6.umin.ac.jp/cgi-open-bin/ctr/ctr_view.cgi?recptno=R000065882.

## Administrative information

Note: the numbers in curly brackets in this protocol refer to SPIRIT checklist item numbers. The order of the items has been modified to group similar items (see http://www.equator-network.org/reporting-guidelines/spirit-2013-statement-defining-standard-protocol-items-for-clinical-trials/).

**Table:** 

Title {1}	“Effects of dual-task training on attentional function among community-dwelling older adults: Study protocol for a randomized controlled trial”
Trial registration {2a and 2b}	UMIN000057681. (30 June 2025) UMIN website https://center6.umin.ac.jp/cgi-open-bin/ctr/ctr_view.cgi?recptno=R000065882
Protocol version {3}	Version 1.0 dated on 30 June 2025
Funding {4}	The study is funded by the Japan Society for the Promotion of Science, KAKENHI under Grant-in-Aid for Scientific Research (C) −24K05416, and Kagamiishi Town, a local government municipality in Japan.
Author details {5a}	Maki Ogasawara, Hiroshi Hayashi, Kazuaki Iokawa, Takaaki Fujita, Koshi Sumigawa, Hironori Kawamata, Iori Kawasaki, Daisuke Matsumoto, Toshimasa Sone
Name and contact information for the trial sponsor {5b}	Investigator-initiated clinical trial; Maki Ogasawara (principal investigator) oga-maki@fmu.ac.jp
Role of sponsor {5c}	This is an investigator-initiated clinical trial. Therefore, the funders played no role in the design of the study; the collection, analysis, and interpretation of data; and the writing of the manuscript.

## Introduction

### Background and rationale {6a}

Through attentional function, individuals select appropriate information from senses, memory, and thoughts. Attentional function forms the basis of various cognitive functions, including orientation, memory, and comprehension [1]. Attentional function is known to peak around the age of 20 and gradually decline with age [2, 3]. This decline leads to difficulties in instrumental activities of daily living (IADLs), such as managing finances [4] and performing laundry [5]. Furthermore, the decline is associated with an increased risk of falls [6, 7] and motor-vehicle accidents [8, 9]. Therefore, preventing the decline of attentional function is a crucial challenge for promoting independent living in older adults.

Dual-task training (DTT) is an intervention designed to enhance attentional control during multitasking. In this training, individuals are required to perform multiple tasks, such as motor and/or cognitive tasks, simultaneously. It has been reported to improve attentional impairments associated with cerebrovascular disorders and traumatic brain injury [10–12]. To maximize the effectiveness of DTT, task difficulty should be individually adjusted based on Dual-Task Interference (DTI) [12, 13]. In DTI, performance deteriorates when two tasks are performed simultaneously, even though each task is manageable when performed alone. Because the effectiveness of DTT is diminished when the level of interference is either too high or too low, an appropriate level should be identified for each individual.

Reviews on DTT for community-dwelling older adults have shown benefits in maintaining their general cognitive function and improving their balance function [14, 15]. However, findings regarding the effects of DTT on attentional function remain inconsistent. Most of the studies that have included attentional function as an outcome measure [16–25] did not report significant improvements, and only three studies [18, 21, 25] demonstrated statistically significant improvements. A common feature of these three studies is that they implemented DTT combining “motor tasks” and “cognitive tasks involving motor activity.” However, four other studies that incorporated similar components [17, 20, 22, 23] did not find improvements in attentional function. Although these studies had no substantial differences in sample sizes (20–50 participants) or intervention durations (1–3 months), inconsistency in outcomes may reflect differences in how task difficulty was appropriately adjusted based on DTI. Moreover, these studies relied on a limited set of measures and comprehensive evaluation of multiple domains of attentional function was lacking. Therefore, studies have not investigated the effectiveness of DTT that combines “motor tasks” and “cognitive tasks involving motor activity,” individually adjusting task difficulty based on DTI for improving attentional function in community-dwelling older adults.

### Objectives {7}

This study aims to determine the effectiveness of a DTT program in enhancing attentional function in community-dwelling older adults. In this DTT program, “motor tasks” and “cognitive tasks involving motor activity” are combined, and task difficulty is individually adjusted based on DTI. This program will be evaluated through a randomized controlled trial (RCT).

### Trial design {8}

The study design is an RCT, in which the DTT is compared to a control group of participants who maintain their usual daily routine. The allocation ratio is 1:1. The superiority of DTT compared to the control group will be tested.

## Methods: participants, interventions, and outcomes

### Study setting {9}

Participants will be recruited from community-dwelling older adults living in Kagamiishi Town, Fukushima Prefecture, Japan. The study will be conducted at the Kagamiishi Town Health and Welfare Center.

### Eligibility criteria {10}

The inclusion criteria will be as follows:

1. Individuals aged 65–80 years
2. Individuals who can complete the Trail Making Test Part A (TMT-A) error-free within 40 to 80 s
3. Individuals who can participate in the intervention twice weekly for four weeks
4. Individuals who have provided informed consent to participate in the study

The age limitation in (1) was applied in consideration of the decline in attentional function with aging. The TMT-A criterion in (2) was based on reference values from the Trail Making Test-Japanese (TMT-J) manual [26], which reports a maximum score of 80 seconds among healthy older adults. Scores below 40 seconds were considered indicative of an adequate attentional function which is unlikely to improve further through intervention. Based on these considerations, the TMT-A criterion was established.

The exclusion criteria will be as follows:

1. Individuals who have been medically advised to restrict physical activities, including those with conditions such as severe hypertension, cardiac diseases, or undergoing treatment for fractures that require exercise limitation.

### Who will take informed consent? {26a}

The principal investigator will explain the study objectives and procedures to all participants and obtain their written informed consent prior to enrollment. Through the participant information, the participants will be informed that all study data will be handled with strict confidentiality.

### Additional consent provisions for collection and use of participant data and biological specimens {26b}

Additional consent will be obtained if the stored data are to be used in a new study upon the completion of the study.

## Interventions

### Explanation for the choice of comparators {6b}

Through the trial, the attentional function of the intervention and control groups at baseline and post-intervention will be assessed to determine the effectiveness of DTT. The control group will consist of community-dwelling older adults living in the same area. They will continue their usual daily activities throughout the study period. As an ethical consideration, participants in the control group will have the opportunity to experience the DTT intervention after the study is complete.

### Intervention description {11a}

The DTT consists of a combination of “motor tasks” and “cognitive tasks involving motor activity”, and task difficulty is individually adjusted based on DTI. The intervention will be conducted at the Health and Welfare Center in Kagamiishi Town. It will be delivered by two occupational therapists who are well-versed in DTT with the assistance of two volunteers for operational support. The intervention will be implemented twice per week for four weeks, with each session lasting one hour. To facilitate implementation in community settings, DTT will be conducted with a group of approximately 30 participants.

Each one-hour session consists of a 5-minute health check, 10-minute warm-up exercises, 15-minute DTT, a 10-minute break, another 15-minute DTT, and a 5-minute cool-down. The specific tasks used in the intervention are presented in Table 1. In the DTT, participants will first practice “motor tasks” and “cognitive tasks involving motor activity” as single tasks. After they are accustomed to performing each task individually, they will perform two tasks simultaneously.

**Table 1:** Examples of DTT task combinations

Motor tasks [18, 21, 22]	Cognitive tasks involving motor activity
・Stepping in a seated position ・Stepping in a standing position	■Finger exercises [21, 25] ∙ Perform combinations of hand gestures, such as rock and paper, rock and scissors, and scissors and paper, with both hands ∙ Perform different hand gestures simultaneously with each hand, such as rock with one hand and paper with the other ∙ Perform complex bilateral movements, alternating between the extension of the thumb on the one hand and the extension of the little finger on the opposite hand
・Stepping in a seated position ・Stepping in a standing position	■Upper limb exercises [17, 18, 21] ∙ Move both upper limbs in the instructed direction ∙ Move both upper limbs in the opposite direction of the instruction ∙ Perform vertical movements with one upper limb and triangular movements with the other ∙ Switch the roles of the upper limbs midway through the task
・Stepping in a seated position ・Stepping in a standing position	■Exercises using tools [18] ∙ Toss and catch a beanbag with one hand ∙ Toss and catch one beanbag with both hands ∙ Toss and catch two beanbags simultaneously with both hands

DTI setting is applied to both tasks. For “motor tasks,” if stepping in a seated position is performed with ease, the task will progress to stepping in a standing position. For “cognitive tasks involving motor activity”, participants will start with low-difficulty tasks, such as simple finger movements, and progress to higher-difficulty tasks when they can easily perform the low-difficulty tasks. Adjustments to task difficulty will be made at the discretion of the occupational therapist. If a task is judged to be too easy for the participant, the therapist will encourage progression to a more challenging level of the task to ensure an appropriate training load. To prevent falls, stepping in a standing position is performed in front of a chair with handrails or other stable supports placed nearby.

### Criteria for discontinuing or modifying allocated interventions {11b}

Criteria for discontinuing the intervention for participants include adverse events that permanently prevent their participation in the intervention.

Discontinuation will be considered in the following cases:

1. Difficulty performing the exercises owing to a significant decline in motor or cognitive function.
2. Inability to participate owing to hospitalization for medical treatment.
3. Physician-imposed medical restrictions on participation owing to outpatient care.

### Strategies to improve adherence to interventions {11c}

Incentives will be provided to the participants to promote their adherence. Participants in the intervention group will receive 500 yen for attending each session. Those who attend all sessions including pre-intervention and post-intervention evaluation (10 out of 10) will receive a total of 5000 yen, while those who attend 8 out of 10 sessions will receive 4000 yen. Participants in the non-intervention group will receive a fixed payment of 5000 yen for completing the post-intervention evaluation.

### Relevant concomitant care permitted or prohibited during the trial {11d}

All types of care will be permitted during the study, other than non-scheduled DTT.

### Provisions for post-trial care {30}

In the event of any health damage to a participant during the study, appropriate treatment will be provided within the scope of public medical insurance coverage. The principal investigator is enrolled in the Occupational Therapist Comprehensive Compensation Insurance System to provide compensation as needed.

### Outcomes {12}

The following outcomes will be assessed at two time points, t0 and t2, as shown in Table 2, and differences in the change scores between the groups will be analyzed.

**Table 2:**
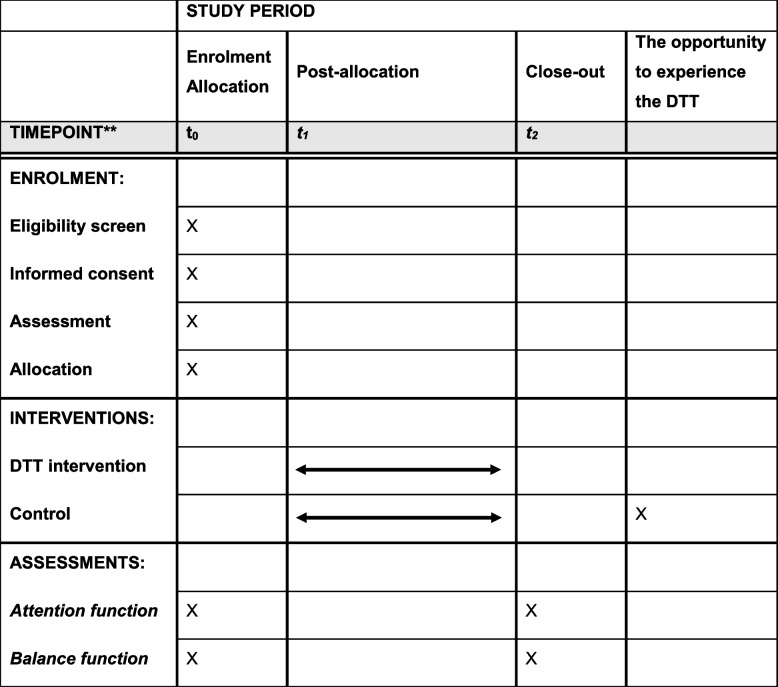
Participant timeline

Time point

t0: baseline

t1: week1–week4

t2: week5

### Primary outcome assessment

To evaluate attentional function, the TMT-J [26] will be used. TMT-J consists of Part A (TMT-A) and Part B (TMT-B). In TMT-A, circles numbered from 1 to 25 are presented, and participants were instructed to quickly connect numbers in ascending order by drawing lines. In Part B (TMT-B), the circles contained either numbers from 1 to 13 or the first 12 characters of the Japanese Hiragana alphabet. Participants are instructed to connect the numbers and characters in alternating order. The time required (in seconds) for each test will be measured and used as an outcome indicator. Additionally, ΔTMT will be calculated by subtracting the time for TMT-A from that for TMT-B (ΔTMT = TMT-B − TMT-A). TMT-A is used to assess sustained attention, while TMT-B is used to evaluate divided attention, with shorter completion times indicating better performance. Additionally, smaller ΔTMT values indicate better executive function. Errors made during the tests will be recorded and included in the analysis as appropriate.

### Secondary outcome assessment

Attentional function will be comprehensively assessed using the digit span test [27, 28], and the Stroop and reverse-Stroop test [29].

The digit span test is a subtest of the Wechsler Memory Scale-Revised (WMS-R), a neuropsychological test designed to assess different memory functions in individuals. It is used to evaluate attention function and attention capacity. Participants are asked to repeat numbers spoken by an examiner in either forward or reverse order. The digit span ranges from three to eight digits, with two trials conducted at each span. If the participant responds correctly on either trial, the task proceeds to the next span. If a participant answers incorrectly twice, the test is terminated. The total score ranges from 0 to 24, with higher scores indicating better performance.

The Stroop and reverse-Stroop test will be used to evaluate the selective attention and attention capacity related to inhibition. The tasks consist of the following four types: (1) Control Condition 1 (selecting color patches corresponding to the meaning of color terms in black ink), (2) Reverse Stroop Condition (selecting color patches corresponding to the meaning of color terms), (3) Control Condition 2 (selecting color terms corresponding to color patches), and (4) Stroop Condition (selecting color terms corresponding to ink colors). Each task will be presented on an A4 paper with 60 trials, and performance will be evaluated based on the number of correct responses within 40 seconds.

To evaluate balance function, the single-leg stance time with eyes open and closed will be measured [30]. This measurement will be taken to assess static standing balance function through an evaluation of whether an individual can maintain balance within a narrow base of support. Further, balance function has been reported to be related to attention [31, 32] and is useful for evaluating physical functions affected by attention. Single-leg stance is measured from the moment a participant’s feet leaves the floor until one of the following occurs: the raised foot touches the floor, the raised foot touches the supporting leg, the position of the supporting foot shifts, or the hands touch the wall. Measurements are conducted for a maximum of 60 seconds, under both eyes-open and eyes-closed conditions. Shorter stance times indicate greater impairment in balance function. When the participants’ eyes are closed, the reduction in their visual input increases the difficulty of the task, often resulting in shorter stance durations than those in the eyes-open condition.

## Participant timeline {13}

### Sample size {14}

We calculated the required sample size as 60 participants based on the results of previous studies on TMT-A and TMT-B [16–18, 21]. We expected a 15-second difference in change scores (post–pre) between the two groups on the TMT-A, with a standard deviation (SD) of 15 seconds. This corresponds to an effect size of 1. Assuming a two-sided α of 0.05 and a statistical power (1–β) of 0.80, the required sample size is 34 participants (17 per group). Next, for TMT-B, we expected a 20-second difference in change scores (post–pre) between the two groups, with a SD of 25 seconds. This corresponds to an effect size of 0.8. Assuming a two-sided *α* of 0.05 and a statistical power (1–β) of 0.80, the required sample size is 52 participants (26 per group). Therefore, the final sample size was determined as 60 participants, considering the potential dropouts.

### Recruitment {15}

Participant recruitment will be conducted by placing advertisements in local media (e.g., public relations publications) and by posting flyers in public spaces within the community. This recruitment strategy has been proven successful in previous projects [33].

## Assignment of interventions: allocation

### Sequence generation {16a}

To ensure equal sample sizes, we will conduct allocation using block randomization. The randomization sequence, with a block size of four, will be produced using computer-generated random numbers (Microsoft Excel, Microsoft Corp., Redmond, WA, USA) by the principal investigator. No stratification of participants will be performed in this study.

### Concealment mechanism {16b}

After the baseline assessment, participants who meet the eligibility criteria will be randomly assigned to the intervention and control groups. The allocator will record the participants’ IDs in an Excel sheet and provide each participant with their group assignment in a sealed envelope. To ensure blinding, recruitment staff, data collectors, and statisticians will remain blinded to the group and will not have access to allocated information.

### Implementation {16c}

The allocation sequence will be generated by the principal investigator, and participant enrolment and intervention assignment will be conducted by a co-investigator.

## Assignment of interventions: blinding

### Who will be blinded {17a}

Owing to the nature of the intervention, participants and those providing the intervention will not be blinded, as group allocation will be evident to them. However, data collectors and the statistician will be blinded.

### Procedure for unblinding if needed {17b}

Not applicable. As this study involves an exercise intervention that is obvious to participants, a procedure for unblinding is not required.

## Data collection and management

### Plans for assessment and collection of outcomes {18a}

All participants will be assessed on all outcome measures at baseline (T0) and at re-evaluation (T2). Prior to data collection, data collectors will receive sufficient training on measurement methods, including the TMT-J, the digit span test, the Stroop and reverse-Stroop test and the single-leg stance time with eyes open and closed. Collected data will be recorded on a record sheet displaying written results, and any missing or inconsistent data will be checked and corrected as necessary. All data will be checked for completeness and accuracy before being entered into a secure electronic database. Before statistical analysis, all data will be cleaned, outliers will be examined, and the normality of the distribution will be tested.

### Plans to promote participant retention and complete follow-up {18b}

The participants who would be unavailable for assessment at the time of the re-evaluation (t2), or who have missing or anomalous data, will be contacted again and offered an opportunity for reassessment. Additionally, participants allocated to the control group will be provided the opportunity to experience the DTT after the completion of the study period.

### Data management {19}

A second investigator will cross-check, and the principal investigator will check again the entered data. A correspondence table linking anonymized IDs will be created and stored in a manner that prevents identification of individuals. When a participant is enrolled in the study, the informed consent form and end-of-trial date will be recorded in secure storage on the university server, and the signed paper forms will be stored in a locked room within the institution. All research data, including participants’ materials, will be archived within the institution. In accordance with institutional regulations, the data will be retained for 10 years after the final publication of study results.

### Confidentiality {27}

Personal data collected from the study participants will be handled in accordance with the Act on the Protection of Personal Information and the standards established by the institutional representative of the research institution. The principal investigator will take all necessary precautions to prevent the leakage, loss, or damage of personal data. Participants’ names written on the questionnaire will be removed during the data transcription and replaced with a research identification (ID) number. The research ID will be a non-identifiable number, beginning with 001.

### Plans for collection, laboratory evaluation and storage of biological specimens for genetic or molecular analysis in this trial/future use {33}

Not applicable. This study will not collect any biological specimens.

## Statistical methods

### Statistical methods for primary and secondary outcomes {20a}

For the primary analysis, the analysis of covariance (ANCOVA) will be performed to compare post-intervention scores between the two groups, with age, sex, and baseline values of each outcome included as covariates. For supplementary analyses, paired t-tests will be conducted to compare pre-intervention and post-intervention means within each group.

The above analyses will be applied to the primary outcomes (TMT-A, TMT-B, and ΔTMT) and secondary outcomes (the digit span test, the Stroop and reverse-Stroop test, and the single-leg stance time).

All analyses will be performed using SPSS Version 29.0 (IBM Corp., Armonk, NY, U.S.) and SAS version 9.4 (SAS Inc., Cary., NC, U.S.).

### Interim analyses {21b}

Not applicable. This study involves a 4-week exercise intervention, and therefore interim analyses were deemed unnecessary.

### Methods for additional analyses (e.g., subgroup analyses) {20b}

Not applicable.

### Methods in analysis to handle protocol non-adherence and any statistical methods to handle missing data {20c}

The primary analysis of the outcome will be conducted using the intention-to-treat principle. Secondary analyses will be performed using the full analysis set and the per-protocol set. Multiple imputations will be conducted for missing values, which will be substituted with the most likely scores using the multiple imputation procedure, resulting in the creation of 10 output datasets.

### Plans to give access to the full protocol, participant level-data and statistical code {31c}

Sharing the participant-level dataset has not been planned. When the primary outcome results are published, the full protocol will also be submitted. However, the datasets analyzed during the current study, the statistical code, and the full protocol will be available from the corresponding author on reasonable request. In such cases, the access of data by a third party will likely require further approval from the ethics committee.

## Oversight and monitoring

### Composition of the coordinating centre and trial steering committee {5d}

This is a single-centre study designed, implemented, and coordinated at Fukushima Medical University. Day-to-day support for the trial is provided by the following staff:

- Principal investigator: Supervises the trial, conducts trial registration and participant enrolment, obtains informed consent, and ensures that the follow-up is conducted in accordance with the protocol.
- Data manager: Organizes data acquisition and safeguards the quality of the data.
- Data collectors: Conduct data acquisition, including assessments of attention and balance functions.
- Statistician: analyzes data after trial completion.

The principal investigator, data manager, data collectors, and statistician will meet before, during, and after the trial.

In this study, no formal independent committees, such as a steering committee or a data monitoring committee, will be established owing to the fact that it is a single-centre study with a low-risk, non-invasive intervention.

### Composition of the data monitoring committee, its role and reporting structure {21a}

Not applicable. In this study, no formal committees will be formed. The funding body has no role in the study design and the collection, analysis, interpretation, and presentation of the study data.

### Adverse event reporting and harms {22}

Adverse events, such as falls and injuries during training, will be recorded. The principal investigator will promptly consult the ethics committee and take appropriate action in response to any serious adverse events, regardless of their causal relationship with the intervention.

### Frequency and plans for auditing trial conduct {23}

Not applicable. In this study, no external audit will be conducted.

### Plans for communicating important protocol amendments to relevant parties (e.g. trial participants, ethical committees) {25}

All protocol modifications will be submitted in writing to the ethics committee for review and approval prior to implementation. Each time an amendment is made, the online trial registry will be updated. All approved protocol amendments will be communicated to relevant stakeholders and investigators.

### Dissemination plans {31a}

The findings of this study, including both positive and negative results, will be published in a peer-reviewed journal. A summary of the research findings will be made available to participants upon request.

## Discussion

### Background and purpose

Attentional function is a fundamental component of various cognitive functions [1], and its decline has been associated with cognitive impairment and difficulties in performing IADLs [4, 5]. Moreover, attention decline is known to affect many aspects of daily life, including an increased risk of falls [6, 7] and a higher risk of motor-vehicle accidents [8, 9]. The above factors show that enhancing attentional function is crucial for enabling community-dwelling older adults to maintain healthy lives in their familiar environments.

Therefore, in the present study, we developed a DTT program with the aim of preventing attention decline and designed a research protocol to evaluate its effectiveness.

### Strengths of this study

The following are the main features of this study.

1. Simplicity and Versatility: The DTT program is designed to be simple and versatile, requiring no large-scale equipment or special facilities, and can be easily implemented in daily life settings.
2. Task Components: The program combines “motor tasks” and “cognitive tasks involving motor activity.” Further, task difficulty will be individually adjusted according to each participant, considering DTI. These two features contribute to an effective task design to enhance attentional function.
3. Comprehensive Assessment of Attention: This study will be conducted to evaluate multiple aspects of attention, including sustained, selective, divided attention, and attentional capacity, using various measures such as TMT-A, TMT-B, digit span test, and the Stroop and reverse-Stroop test. These comprehensive assessments will be used to enable a more detailed examination of the effects of DTT on attentional function.

### Future directions

The DTT program developed in this study can be implemented regardless of time and place. In the future, its dissemination and integration into community gatherings and preventive care services for older adults may be promoted through brochures and instruction manuals. This approach is expected to promote the maintenance and improvement of attentional function among community-dwelling older adults, ultimately contributing to the extension of healthy life expectancy and the enhancement of their quality of life.

## Trial status

Trial registration: UMIN, UMIN000057681. (Registered 30 June 2025).

UMIN website https://center6.umin.ac.jp/cgi-open-bin/ctr/ctr_view.cgi?recptno=R000065882

(Version 1.0 dated 30 June 2025)

The data recruitment began in August 2025, and recruitment will be completed on 25 September 2025.

## Data Availability

The authors will have access to the final trial dataset. Statisticians responsible for outcome analysis will be provided with the complete dataset once data collection is complete. Data required to support the protocol will be made available upon request. However, access to the data by individuals who have not been approved by the ethics committee may require additional ethical approval.

## Abbreviations

DTI: Dual-task interference
DTT: Dual-task training
TMT-J: Trail Making Test-Japanese
TMT-A: Trail Making Test-Japanese part A
TMT-B: Trail Making Test-Japanese part B
IADLs: Instrumental activities of daily living
